# Simultaneous dual-channel imaging to quantify interdependent protein recruitment to laser-induced DNA damage sites

**DOI:** 10.1080/19491034.2018.1516485

**Published:** 2018-10-20

**Authors:** Joachim Garbrecht, Harald Hornegger, Sebastien Herbert, Tanja Kaufmann, Josef Gotzmann, Kareem Elsayad, Dea Slade

**Affiliations:** a Department of Biochemistry, Max F. Perutz Laboratories, University of Vienna, Vienna Biocenter (VBC), Vienna, Austria; b Department of Medical Biochemistry, Max F. Perutz Laboratories (MFPL), Medical University of Vienna, Vienna Biocenter (VBC), Vienna, Austria; c VBCF-Advanced Microscopy, Vienna Biocenter (VBC), Vienna, Austria

**Keywords:** Laser-induced microirradiation, simultaneous imaging, PARP1, PARG, PCNA, ALC1

## Abstract

Fluorescence microscopy in combination with the induction of localized DNA damage using focused light beams has played a major role in the study of protein recruitment kinetics to DNA damage sites in recent years. Currently published methods are dedicated to the study of single fluorophore/single protein kinetics. However, these methods may be limited when studying the relative recruitment dynamics between two or more proteins due to cell-to-cell variability in gene expression and recruitment kinetics, and are not suitable for comparative analysis of fast-recruiting proteins. To tackle these limitations, we have established a time-lapse fluorescence microscopy method based on simultaneous dual-channel acquisition following UV-A-induced local DNA damage coupled with a standardized image and recruitment analysis workflow. Simultaneous acquisition is achieved by spectrally splitting the emitted light into two light paths, which are simultaneously imaged on two halves of the same camera chip. To validate this method, we studied the recruitment of poly(ADP-ribose) polymerase 1 (PARP1), poly (ADP-ribose) glycohydrolase (PARG), proliferating cell nuclear antigen (PCNA) and the chromatin remodeler ALC1. In accordance with the published data based on single fluorophore imaging, simultaneous dual-channel imaging revealed that PARP1 regulates fast recruitment and dissociation of PARG and that in PARP1-depleted cells PARG and PCNA are recruited with comparable kinetics. This approach is particularly advantageous for analyzing the recruitment sequence of fast-recruiting proteins such as PARP1 and ALC1, and revealed that PARP1 is recruited faster than ALC1. Split-view imaging can be incorporated into any laser microirradiation-adapted microscopy setup together with a recruitment-dedicated image analysis package.

## Introduction

Laser microirradiation in combination with live cell imaging is commonly used for studying the recruitment of proteins involved in DNA damage response [1–5]. Imaging real-time recruitment of fluorescently-tagged proteins to sites of laser-induced DNA damage enables analysis of the spatiotemporal dynamics of the recruitment process [6]. DNA lesions are usually induced as spots or stripes of laser-microirradiated regions across a nucleus. Damage can be induced using in-built lasers (e.g., 405 nm lasers targeted to cells in the FRAP mode of laser-scanning confocal microscopes) or add-on laser modules, such as UV lasers and near infrared (800 nm) multiphoton lasers [3]. Induction of DNA damage using low-energy UV-A lasers (315–400 nm) is facilitated by presensitization with DNA-intercalating Hoechst 33258 and 33342 dyes or with a nucleoside analogue 5-bromo-2ʹ-deoxyuridine (BrdU), which obviate the need for high laser intensity [1,4]. UV-A laser light typically generates UV-type lesions such as cyclobutane pyrimidine dimers (CPDs) and 6–4 photoproducts (6–4PPs), as well as oxidative base damage and abasic sites due to reactive oxygen species produced in the aqueous cellular environment [7,8]. UV-A laser is thus particularly suitable for studying base excision repair (BER) [3]. Presensitization facilitates single-strand breaks (SSBs) and double-strand breaks (DSBs) upon UV activation, and thus enables the study of proteins involved in single-strand break repair (SSBR) and double-strand break repair (DSBR) as well [1–4,9]. Near infrared high-energy lasers generate a broad spectrum of DNA lesions but without the need for presensitization [4,5,10,11].

High-speed imaging configuration enables analysis of the recruitment and dissociation kinetics immediately after DNA damage induction, which is essential for characterization of the dynamic behaviour of proteins involved in DNA damage response. Compared to traditional approaches to studying DNA-damage induced recruitment by immunofluorescence imaging of ionizing radiation-induced foci (IRIF) or chromatin-immunoprecipitation (ChIP), the microirradiation imaging system offers two distinct advantages: (i) the formation of highly localized tracks of laser-induced lesions within the nucleus versus homogeneous distribution of DNA damage induced by ionizing radiation, which enables visualization of proteins that do not form IRIF, and (ii) visualization of recruitment immediately after DNA damage induction through short-interval imaging [5].

Kinetics of protein recruitment to laser-induced DNA damage sites may vary depending on the laser type, laser power and presensitization methods (BrdU, Hoechst, or both), all of which influence the type of damage that is generated within DNA, as well as the type of cell line (most commonly used are U2OS and HeLa), fluorescent tags and protein expression levels [3,5,11,12]. Comparative analysis of recruitment kinetics revealed a general agreement between the temporal protein recruitment sequence and the timing of the respective step in DNA damage response in which the protein exerts its function; DNA damage-sensing proteins are recruited first, followed by proteins involved in early steps of DSB repair and chromatin remodelers, whereas proteins involved in DNA damage signalling and homologous recombination exhibit a broader range of recruitment timing [8,12].

Hitherto published fluorescence microscopy methods are dedicated to the study of single fluorophore (and hence single protein) kinetics by live imaging or use sequential dual-fluorophore imaging for studying two proteins. Although these methods provide a good measurement of a single protein recruitment, they may not be suitable for comparing two or more proteins due to large cell-to-cell biological variability in gene expression and, consequently, protein concentration. In order to overcome this, a large number of samples needs to be analysed to yield statistically significant correlations. Additionally, averaging large populations, without taking into account possible cross-correlations between the proteins of interest, may mask weaker or more complex recruitment kinetics and the local effects of protein concentration or environment. Perhaps the major drawback of standard single-fluorophore imaging techniques is their ineffectiveness in distinguishing the recruitment sequence of fast-recruiting proteins.

To compare recruitment dynamics of two proteins, we set up simultaneous dual-channel acquisition of two fluorescently tagged proteins following UV-A-induced local DNA damage (355 nm laser with BrdU presensitization), and developed a standardized image and recruitment analysis workflow. To validate our method we used four proteins involved in DNA damage response: poly(ADP-ribose) polymerase 1 (PARP1), poly (ADP-ribose) glycohydrolase (PARG), proliferating cell nuclear antigen (PCNA) and Amplified in Liver Cancer 1 (ALC1). PARP1 synthesizes poly(ADP-ribose) (PAR) from NAD, while PARG removes PAR by cleaving glycosidic bonds between ADP-ribose units [13]. PCNA is a ring-shaped clamp around DNA that serves as a loading platform for proteins involved in DNA replication and repair [14]. ALC1 is an SNF2-type chromatin remodeling ATPase, which is activated upon PAR binding to its macro domain at DNA damage sites [15,16]. PARP1, PARG, PCNA and ALC1 are all recruited to laser-induced DNA damage sites albeit with distinct recruitment kinetics.

PARP1 is rapidly and transiently recruited to DNA damage sites reaching a maximum about 1 minute on average after microirradiation [17]. Its initial recruitment is mediated by the Zn-finger DNA-binding domain and by PAR-binding, while its dissociation is dependent on its catalytic activity [17]. DNA-damage dependent activation of PARP1 represents an early response to genotoxic stress with PARP1 often referred to as the sensor of DNA strand breaks [18]. The first catalytic target is the auto-modification domain of PARP1 itself, followed by PARylation of histones and other proteins at DNA damage sites [19,20]. PARP1 promotes chromatin relaxation at DNA damage sites by PARylating and displacing histone H1 [21,22] and recruiting chromatin remodelers such as ALC1 [15,16,23], SMARCA5 [24] and CHD2 [25]. Chromatin relaxation allows access to DNA repair factors, some of which are recruited through binding PAR. PARP1 mediates the initial accumulation of MRE11–RAD50–NBS1 (MRN) complex at DNA lesions and promotes rapid recruitment of BRCA1, BRCA2 and EXO1 involved in homologous recombination [26–29]. PAR also promotes recruitment of XRCC1 as a scaffold protein in base excision repair [17,30,31] and XPA as a scaffold protein in nucleotide excision repair [32].

Unlike PARP1, PCNA is slowly recruited to DNA damage sites reaching maximum intensity 4–6 min after microirradiation [33,34]. PCNA remains stably bound at DNA damage sites to coordinate timely recruitment of DNA replication and repair factors [6,33,34]. As a ring-shaped homotrimer, PCNA encircles and slides along DNA, and recruits proteins via the interdomain connector loop on the outer surface serving as the common PCNA-protein interaction interface [14]. Structure-specific endonuclease ZRANB3, exonuclease EXO1, DNA methyl transferase DNMT1, replication licensing factor CDT1, PCNA loader RFC1 and PARG bind PCNA via the PCNA-interacting protein motif (PIP-box), which also mediates their recruitment to laser-induced DNA damage sites in a PCNA-dependent fashion [35–42]. In the case of EXO1 and PARG, PARP1 promotes early recruitment, while PCNA is responsible for their retention at DNA damage sites [36–38].

Rapid PAR-dependent recruitment to laser-induced DNA lesions is followed by rapid dissociation to ensure transient association of proteins with DNA damage sites, unless their sustained residence is required for DNA repair (as in the case of EXO1 [38]). By cleaving PAR, PARG promotes timely protein dissociation from DNA damage sites, as shown in the case of BRCA1, XRCC1, CHD2 and TRIM33 [25,26,43,44].

Using simultaneous imaging of PARP1, PCNA, PARG or ALC1 we confirmed previously published recruitment kinetics of these proteins and revealed additional aspects of their relative recruitment dynamics.

## Results

### Image processing of simultaneous protein recruitment to damage sites

**Figure 1.**
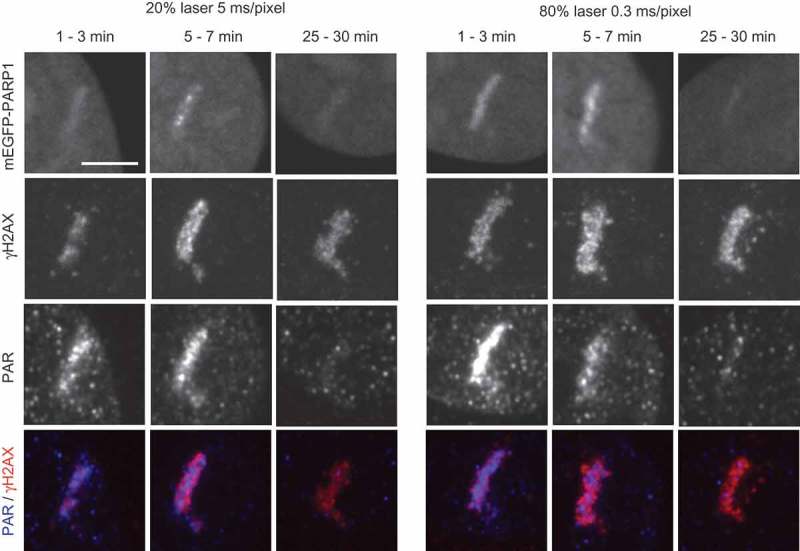
**DNA damage induction with a UV-A 355 nm laser coupled with BrdU presensitization**. U2OS cells were transfected with mEGFP-PARP1, presensitized with 10 μM BrdU for 16 h and exposed to laser microirradiation along a defined stripe. Two UV laser settings were used: 20% laser intensity, 5 ms/pixel pulse duration or 80% laser intensity and 0.3 ms/pixel pulse duration. Immunofluorescence images show DNA damage markers PAR and γH2AX at different time points after laser-induced DNA damage. Image acquisition settings and brightness/contrast adjustments were the same between time points and conditions to allow direct comparison of the stainings. Scale bar = 5 μm.

**Figure 2.**
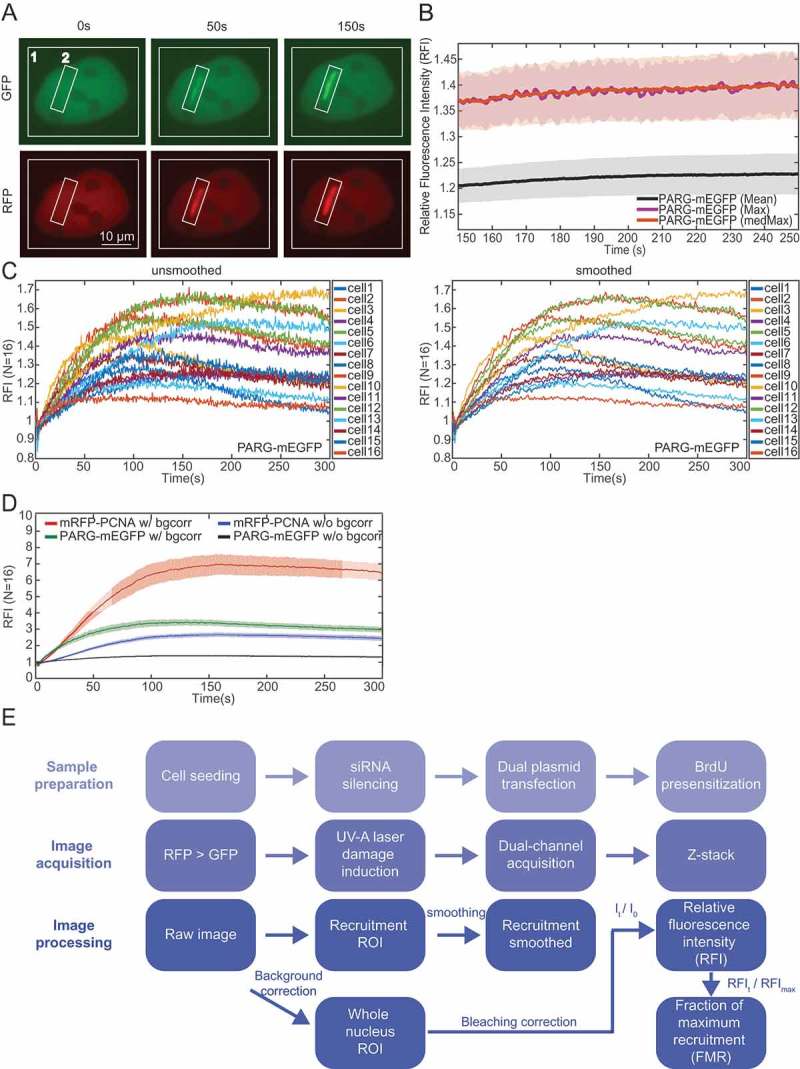
**Image processing pipeline**. a) Time-lapse images of U2OS cells transfected with PARG-mEGFP and mRFP-PCNA. ROI marked ‘1ʹ is used for bleaching correction; ROI marked ‘2ʹ for damage quantification. b) Comparison of quantification methods. Median of the 20 maximum intensities (‘medMax) shows lower noise without significant amplitude shift compared to the maximum value (‘Max’). Relative fluorescence intensity (RFI = I_t_/I_0_) is shown. c) Comparison of unsmoothed lines (left panel) and after applying a smoothing factor of three (right panel) shown for PARG-mEGFP. The smoothing factor was calculated by averaging the intensity at each time point *I*(x,t_i_)) with that of the immediately preceding (*I*(x,t_i-1_)) and the subsequent time point (*I*(x,t_i+1_)). Smoothing reduces noise on a single cell level without affecting recruitment. d) The effect of background correction. Background correction changes the amplitude without changing kinetics. e) The workflow of simultaneous dual-channel imaging and image analysis. All error bars show mean +/- SEM.

**Figure 3.**
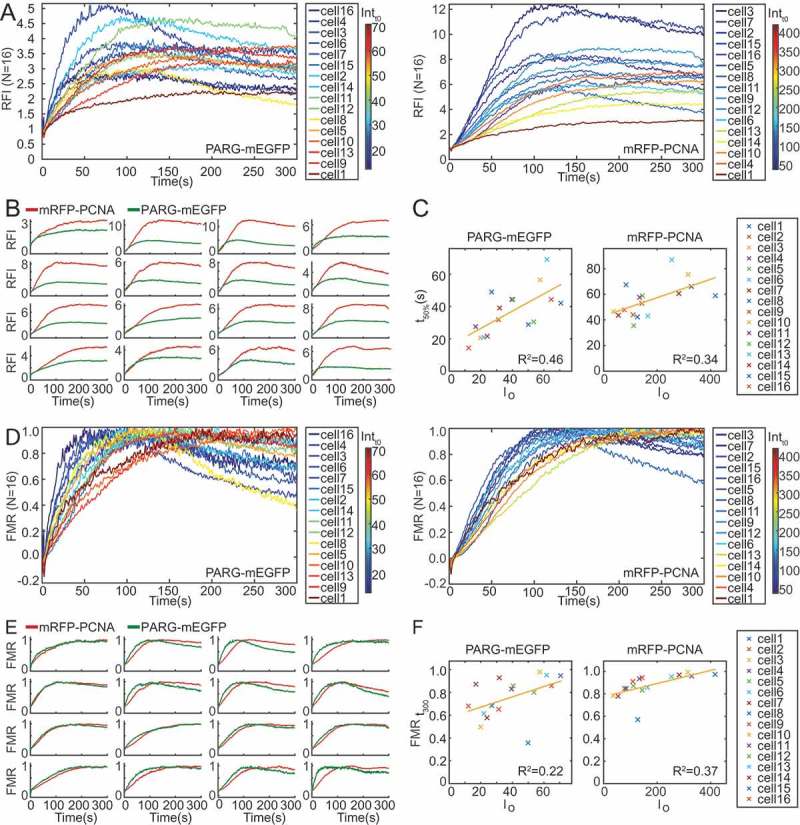
**Data analysis pipeline**. a) Single cell recruitment of PARG-mEGFP (left panel) and mRFP-PCNA (right panel), colour coded according to initial fluorescence intensity. b) Dual-channel recruitment on a single cell level. mRFP-PCNA shows higher fold change than PARG-mEGFP in all cells. c) Scatter plot of the time point when 50% of maximum RFI was reached relative to the initial fluorescence intensity. Linear regression analysis yields R^2^ = 0.46 for PARG-mEGFP and R^2^ = 0.34 for mRFP-PCNA. d) Fraction of maximum recruitment (FMR) shown for individual cells. For PARG-mEGFP (left), two different kinetic trends with partial correlation with initial intensity are distinguishable: in cells with lower initial intensities maximum recruitment is reached faster followed by faster dissociation; high-intensity cells exhibit slower recruitment without dissociation during the 300 s of imaging. This effect is less prominent for mRFP-PCNA (right). e) Dual-channel FMR on a single cell level. PARG-mEGFP shows faster initial recruitment compared to mRFP-PCNA in all cells. f) Scatter plot of the fraction of maximum recruitment (FMR) after 300 seconds relative to the initial intensity. Linear regression analysis yields R^2^ = 0.22 for PARG-mEGFP and R^2^ = 0.37 for mRFP-PCNA.

**Figure 4.**
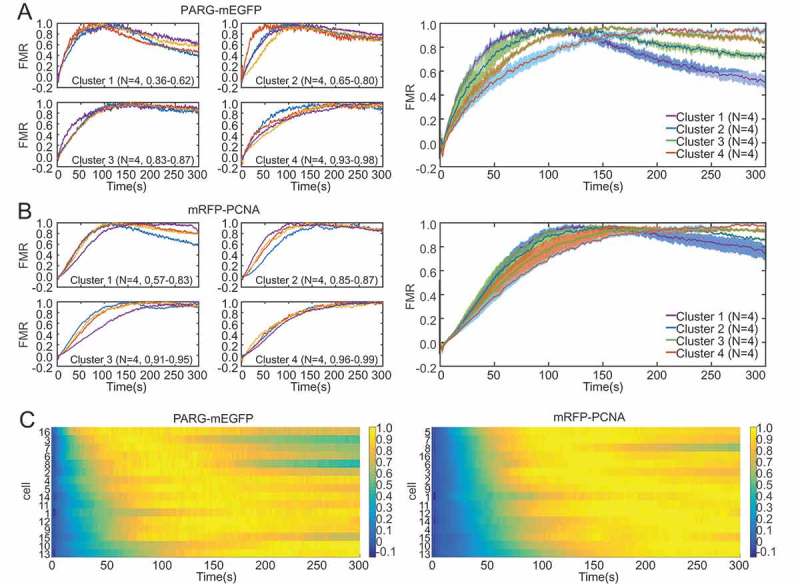
**Data clustering pipeline**. a) Clustering was performed by grouping the cells into four equally sized clusters according to the FMR value after 300 seconds. The range of FMR values in each cluster is indicated in brackets. Clustering shows homogeneity inside clusters (left panel) and helps identify two subpopulations (right panel). b) Clustering of mRFP-PCNA (left) results in subpopulations that are less distinct compared to PARG-mEGFP (right panel). c) Heatmaps represent fraction of maximum recruitment with blue being lowest and yellow being highest for PARG-mEGFP (left) and mRFP-PCNA (right). Cells were ordered according to initial recruitment and show correlation between strong initial recruitment and early dissociation. Error bars represent mean +/-SEM.

**Figure 5.**
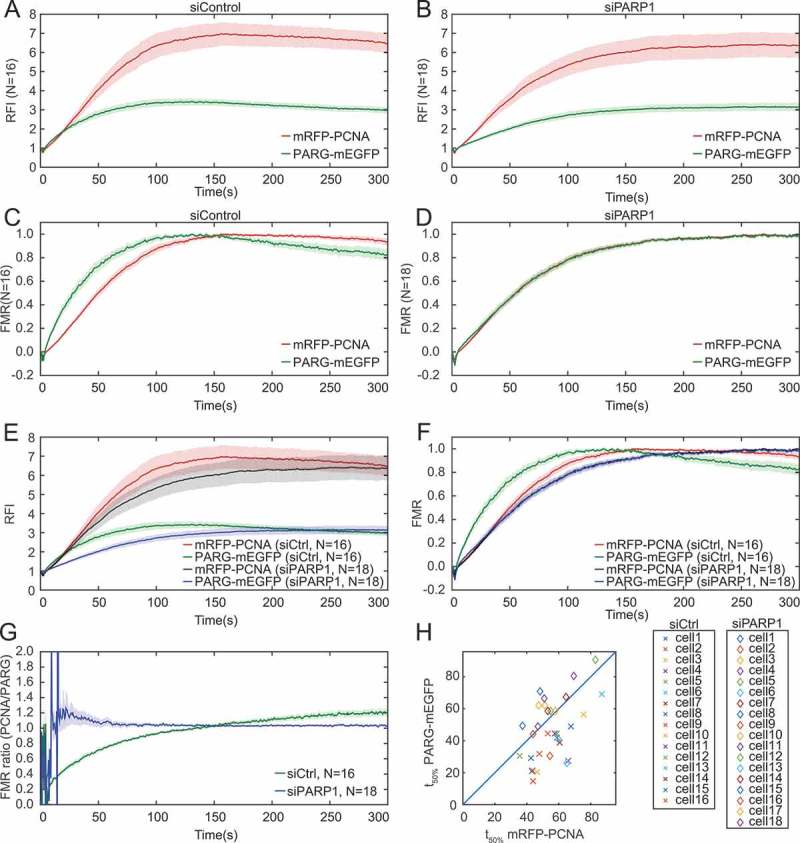
**PARG and PCNA are recruited with comparable kinetics when PARP1 is silenced**. a, b, e) Relative fluorescence intensity (RFI) and c, d, f) fraction of maximum recruitment (FMR) for a, c) siControl-, b, d) siPARP1-transfected cells and e, f) siControl and siPARP1 cells expressing PARG-mEGFP and mRFP-PCNA. In siControl cells PARG shows faster recruitment while PCNA shows a bigger fold change. PARP1 silencing results in comparable recruitment profiles for PARG and PCNA. g) Mean ratio of FMR for mRFP-PCNA and PARG-mEGFP on a single cell level shows a plateau at very early times for siPARP1. Initial noise signal is due to relatively strong noise prior to recruitment. h) Scatter plot of the time of 50% of maximum recruitment for PARG-mEGFP vs mRFP-PCNA. Error bars represent mean +/- SEM.

**Figure 6.**
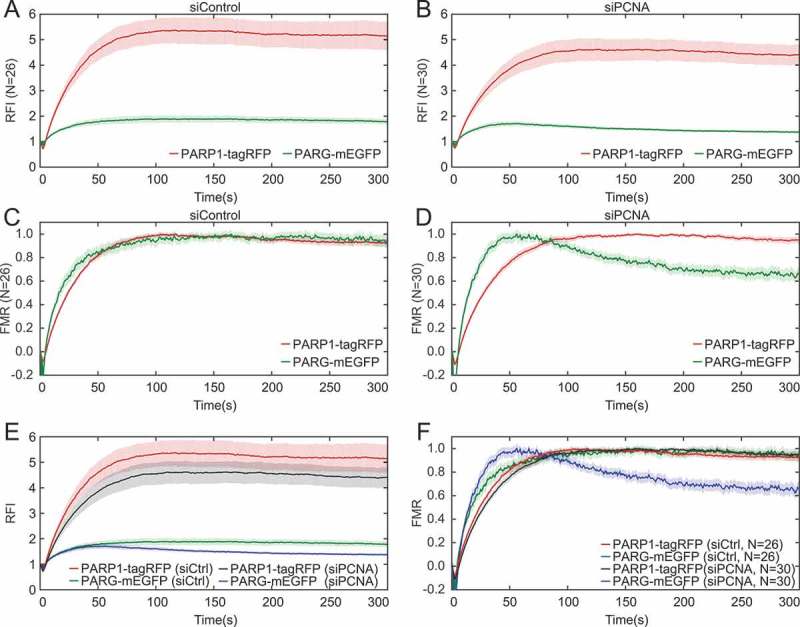
**PCNA stabilizes PARG at DNA damage sites**. a, b, e) Relative fluorescence intensity (RFI) and c, d, f) fraction of maximum recruitment (FMR) for a, c) siControl-, b, d) siPCNA-transfected cells and e, f) siControl and siPCNA cells expressing PARG-mEGFP and PARP1-tagRFP. In siControl cells PARG and PARP1 remain bound to DNA damage sites during 300 s of imaging. PCNA silencing results in PARG dissociation from damage sites, without affecting PARP1 recruitment kinetics.

**Figure 7.**
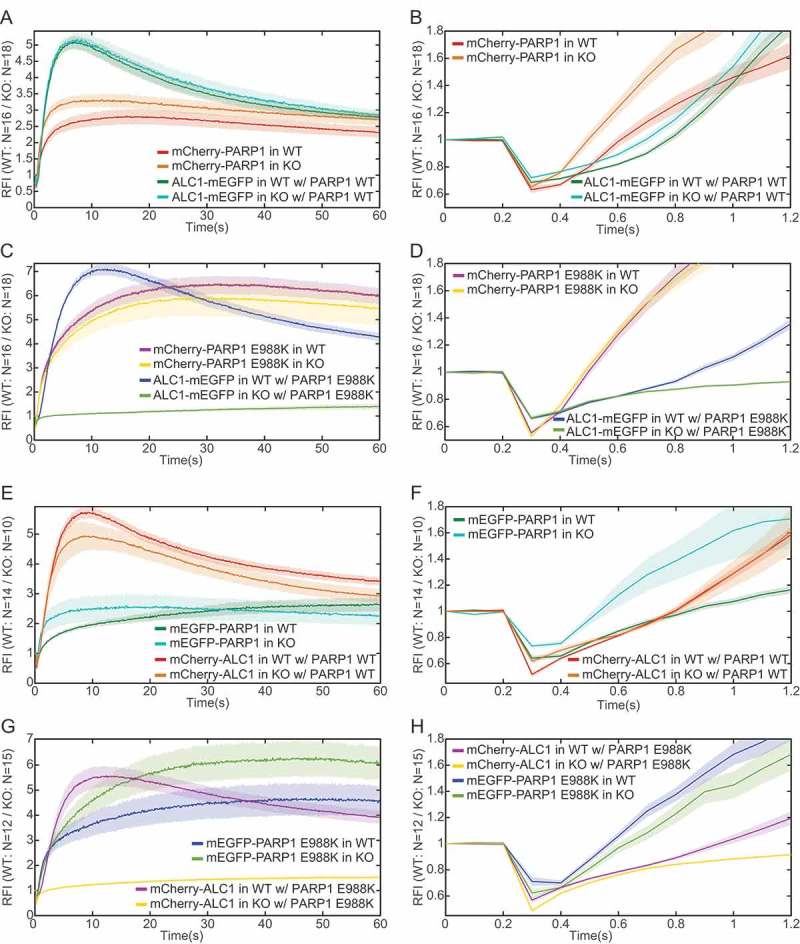
**Dual-channel imaging allows distinction in kinetics between fast-recruiting proteins PARP1 and ALC1**. Relative fluorescence intensity (RFI) of a, b) ALC1-mEGFP and mCherry-PARP1 in PARP1 WT and KO cells, c, d) ALC1-mEGFP and mCherry-PARP1 E988K in PARP1 WT and KO cells, e, f) mEGFP-PARP1 and mCherry-ALC1 in PARP1 WT and KO cells, and g, h) mEGFP-PARP1 E988K and mCherry-ALC1 in PARP1 WT and KO cells during a, c, e, g) 60 s or b, d, f, h) 1.2 s of imaging after laser-induced DNA damage. Image processing was performed without smoothing. Error bars represent mean +/- SEM.

The type and the amount of DNA damage induced by laser microirradiation is known to depend on the laser type, intensity and the use of presensitizers [3,5,11,12]. U2OS cells were presensitized with BrdU and 355 nm UV-A laser was applied in two settings: (i) at 20% laser intensity (130 μW at the sample) and laser pulse duration of 5 ms/pixel (for recruitment analysis shown in Figure 2–6) and (ii) at 80% laser intensity (820 μW at the sample) and laser pulse duration of 0.3 ms/pixel (for recruitment analysis shown in Figure 7). DNA damage induced by the two different laser settings was analyzed in U2OS cells transiently transfected with mEGFP-PARP1 to facilitate identification of damaged cells for subsequent immunofluorescence analysis of PAR and γH2AX generated along the laser path (Figure 1). Both laser settings induced fast and transient recruitment of PARP1, coupled with transient accumulation of PAR, in contrast to sustained accumulation of γH2AX, which persisted > 30 min after irradiation (Figure 1). While PARP1 recruitment and PAR production may be used as a marker of both SSBs and DSBs [29,34], phosphorylation of the histone variant H2AX (γH2AX) is primarily induced at DSBs and is known to spread megabases away from the damage site and persist beyond the time required for DSB repair [45,46]. Given that PAR and γH2AX do not exhibit the same distribution along the laser stripe (Figure 1, bottom overlay panels), both SSBs and DSBs seem to be formed under our experimental conditions.

In order to monitor recruitment of two different proteins to laser-induced DNA damage sites, U2OS cells were co-transfected with two plasmids expressing mEGFP- and m/tagRFP- or mCherry-tagged proteins. Split-view time-lapse imaging was performed on a widefield microscope using OptoSplit II, which enables simultaneous acquisition of two images with different emission colours onto the same camera chip (Figure 2a). Images were acquired every 0.5 s for a period of 300 s. Multiple steps in image processing and analysis will be presented using simultaneous imaging of PARG-mEGFP and mRFP-PCNA as an example (overview in Figure 2e).

The first step in image processing was ‘difference of Gaussians’ (DoG) filtering in Fiji with variance values of 65 nm and 13 µm to reduce the salt and pepper high frequency noise as well as out of focus emission and field of view (FOV) excitation heterogeneity. After filtering, histograms of the fluorescence intensity of the damaged area and the entire nucleus were extracted during the whole acquisition. Damaged area was selected as a rectangular region covering also a small portion of the non-damaged area to account for subtle cellular movement during acquisition (Figure 2a). In order to minimize photobleaching and phototoxicity induced by the sample excitation, we used the lowest possible excitation power. As a consequence, signal to noise ratios (SNR) were low. We used the median of the 20 highest intensities (‘medMax’ in Figure 2b) to assess recruitment, as despite DoG filtering the maximum values can be subject to significant salt-and-pepper type noise. The average intensity of the damaged area (‘mean’ in Figure 2b) was not used given that the selected region of interest also comprises the non-damaged area as shown in Figure 2a. However, it should be noted that chromatin relaxation might also lead to a decrease of the maximum value [47].

Data was smoothed by a factor of three by averaging the intensity at each time point (*I*(x,t_i_)) with that of the immediately preceding (*I*(x,t_i-1_)) and the subsequent time point (*I*(x,t_i+1_)) in order to further reduce noise (Figure 2c). Furthermore, photobleaching was corrected by dividing measured fluorescence intensity with the average nucleus fluorescence for each time point (compare ‘w/bgcorr’ to ‘w/o bgcorr’ in Figure 2d). Two approaches are available for quantifying protein recruitment to DNA damage sites while taking into account unavoidable differences in protein expression levels among cells. In the first approach, the initial intensity (I_0_) is subtracted from the measured intensity at each time point (I_t_-I_0_). This is based on the assumption that the initial fluorescence intensity does not affect the amount of protein being recruited to a damage site. Second, fluorescence intensity at any given time point (I_t_) can be normalized against initial intensity prior to damage (I_0_), yielding relative fluorescence intensity $\left(RFI=\frac{I_{t}}{I_{0}}\right)$ (Figure 2d). In addition to RFI, we also determined the relative kinetics of protein recruitment as explained below.

### Image analysis of simultaneous protein recruitment to damage sites

We developed a standardized image analysis pipeline to investigate several aspects of protein recruitment to DNA damage sites. For population-based analysis, the recruitment profiles for a given protein in a cell population were visualized by plotting RFI of each channel from different cells (Figure 3a). For cell-by-cell analysis, both channels were plotted together to examine simultaneous recruitment of two proteins at a single cell level (Figure 3b). Higher initial intensities for the red channel were chosen in order to minimize the bleed-through effect from the green channel (compare the initial intensity values I_0_ for the two channels in Figure 3a). The initial fluorescence intensity of each cell was colour coded to investigate the large amplitude range between individual cells (Figure 3a). For some cells we noticed an influence of the initial fluorescence intensity on the amplitude of recruitment; cells with lower initial intensity recruited and dissociated faster, while cells with higher intensity showed comparatively delayed recruitment and dissociation (Figure 3a). To investigate the effect of the initial intensity on protein recruitment in more detail, we compared initial intensity to the time point at which a protein reaches 50% of its maximum RFI (Figure 3c). Based on the linear regression analysis, there is no correlation between the initial intensity and the recruitment kinetics for either PARG-mEGFP or mRFP-PCNA (R^2^ < 0.5). This applies to the simultaneous recruitment scenario (Figure 3) as well as when investigating individual recruitment kinetics (Supplementary Figure 2). As a result, we could not determine a range of initial intensities with homogenous recruitment profiles. It should be noted, though, that in the low intensity range the heterogeneity of recruitment kinetics was more pronounced. To minimize the influence of initial intensity on recruitment kinetics, comparable intensity ranges between conditions were used and a large number of cells was analyzed for generating average recruitment profiles, resulting in reproducibility throughout acquisitions.

To analyze recruitment kinetics independent of the amplitude, we calculated the fraction of maximum recruitment (FMR) by rescaling RFI from 0 to 1, 0 being RFI at t_0_ and 1 being maximum RFI (Figure 3d,e). This type of analysis proved very useful for investigating the kinetic mechanism of protein recruitment. We could observe two populations, which again did not show linear correlation with initial intensity when compared to the fraction of maximum recruitment at the last measured time point (Figure 3f and Supplementary Figure 2B). The existence of two distinct populations was particularly prominent for PARG-mEGFP (left panel in Figure 3d). Importantly, the existence of different subpopulations would be lost in standard kinetic analyses due to averaging.

We analyzed this further by clustering the cells according to the last rescaled RFI value (t = 300 s) into four clusters (Figure 4a,b). In the case of PARG-mEGFP we confirmed the existence of two subpopulations with different recruitment dynamics (Figure 4a). The two subpopulations, though less pronounced, were also identified for mRFP-PCNA (Figure 4b). For better presentation we generated a heatmap display, whereby FMR values for each cell were displayed throughout the course of recruitment with a colour code, blue indicating lowest FMR values and yellow indicating highest. In addition, the cells were sorted according to the mean FMR value between time point 15 and 150 (Figure 4c). This type of display confirmed the existence of two distinct populations, especially in the case of PARG-mEGFP-expressing cells, where maximum recruitment was reached faster followed by faster dissociation (top cluster in the left panel of Figure 4c).

We tested whether two PARG or PCNA subpopulations could be linked with nuclear size as an indirect marker of the cell cycle stage. To this end we plotted the size of each nucleus to the time point when a given protein reaches 50% of its maximal recruitment (Supplementary Figure 3). We could not observe a linear correlation between the nuclear size and the recruitment kinetics of PARG and PCNA.

In addition, we performed a cross-correlation analysis to investigate whether the initial fluorescence intensity of one protein may affect the recruitment kinetics of the other (Supplementary Figure 4). Linear regression analysis revealed no cross-correlation between the initial intensity and the recruitment kinetics for PARG and PCNA (R^2^ < 0.6).

### PARP1 and PCNA promote PARG recruitment and residence at DNA damage sites

To validate our imaging and analysis approach, we used three proteins: PARG, PARP1 and PCNA. PARG is recruited initially through PARP1 and stabilised by PCNA [36,37]. PCNA and PARG form characteristic replication foci in S-phase cells, but exhibit a diffuse pattern in G1 and G2 phase of the cell cycle [36,37]. As replication foci were found to interfere with accurate image analysis, we restricted our analysis to cells lacking foci.

PARP1 was silenced in cells in which PARG-mEGFP and mRFP-PCNA were transiently co-expressed (Figure 5). In control cells, PARG and PCNA were rapidly recruited to DNA damage sites, reaching maximum recruitment on average after 100 and 150 s, respectively (Figure 5a). PARG showed faster initial recruitment and faster dissociation compared to PCNA (Figure 5c). While PARP1 silencing had negligible effects on PCNA recruitment, the rate of PARG association to and dissociation from DNA damage sites was reduced (Figure 5a-f). As a result, the mechanism of PARG and PCNA recruitment was mutually indistinguishable in PARP1-depleted cells (Figure 5d, f).

Using dual-channel imaging we can further distinguish whether the observed phenomena are due to an averaging effect or occur at a single cell level. To this end, we determined the ratio between the rescaled red (mRFP-PCNA) and green (PARG-mEGFP) FMR on a single cell level and compared the average of the ratios between siControl and siPARP1 conditions. Within 15 s after laser-induced DNA damage the average of the ratios reached a plateau in the silenced population, while control cells exhibited a continuous increase of the red/green ratio (Figure 5g). Simultaneous imaging also showed that both proteins reach their 50% maximum at comparable time points when PARP1 is silenced (Figure 5h). We thus conclude that similar recruitment kinetics of PARG and PCNA in the absence of PARP1 is not an averaging artefact but occurs on a single cell level.

We also evaluated the effect of PCNA silencing on PARP1 and PARG in cells expressing PARP1-tagRFP and PARG-mEGFP (Figure 6). In control cells, PARP1 and PARG reached maximum recruitment levels at 100 and 120 s respectively (Figure 6a) and showed a similar recruitment mechanism (Figure 6c). While PCNA silencing did not affect the recruitment mechanism of PARP1, it accelerated PARG dissociation from DNA damage sites (Figure 6b,d-f).

Taken together, our results confirm that (i) the initial PARG recruitment is PARP1-dependent and faster relative to PCNA, and that (ii) PCNA promotes PARG stabilization at DNA damage sites [36,37], and reveal that (iii) in the absence of PARP1, PARG and PCNA are recruited with the same kinetic mechanism. Reproducing earlier findings validates our imaging method and analysis approach, while adding mechanistic details to the relative recruitment dynamics of PARG and PCNA in the presence and absence of PARP1.

### Simultaneous imaging to determine the recruitment sequence of two fast-recruiting proteins, PARP1 and ALC1

Simultaneous imaging of protein recruitment to laser-induced DNA damage sites may be particularly advantageous in the case of fast-recruiting proteins, where even subtle variations in recruitment kinetics among cells would preclude direct comparison of two proteins when analyzed in different cells or in a sequential regimen. Thus we compared early recruitment dynamics of PARP1 and ALC1, both of which are known as fast recruiters with maximum recruitment reached within < 1 min [15–17,29,48,49] (Figure 7).

In previous experiments, the 355 nm UV laser was at 20% of full power and irradiation speed of 5 ms/pixel. Using a stripe length of 100 pixels the damage induction procedure lasted for ~ 1.5 s due to the mechanical characteristics of the laser setup. As a result, fluorescently tagged molecules that were localized near the site of damage were bleached throughout this process as well as those that were recruited during this time, which prohibited recruitment analysis at early time points. In order to analyze early recruitment, we decreased irradiation time to 0.3 ms/pixel and reduced the stripe length to 60 pixels. To induce a similar amount of damage, laser intensity had to be increased to 80% (Figure 1). Additionally, we also decreased the exposure time to achieve higher temporal resolution. To counteract the resulting loss of signal-to-noise ratio, we adjusted the excitation intensity to 80% in the green and to 64% in the red emission channels. To maintain an acceptable signal-to-noise ratio, the exposure time of 50 ms with 100 ms interval was used.

To compare early recruitment of ALC1 and PARP1, we used a combination of mEGFP- and mCherry-tagged proteins, as these fluorophores have comparable bleaching behaviour (Supplementary Figure 5). We focused on the first 1.2 s of the time lapse series (Figure 7b, d, f, h). DNA damage was induced after having acquired three frames (200 ms). When using a combination of mCherry-PARP1 and ALC1-mEGFP, PARP1 showed visibly faster recruitment (red and green curves in Figure 7b). However, in the case of mEGFP-PARP1 and mCherry-ALC1 the difference was less pronounced (red and green curves in Figure 7f). These differences were not due to initial fluorescence intensity as individual cells showed comparable recruitment profiles for a given protein despite variations in initial intensity (Supplementary Figure 6A,B). They were also not due to the initial intensity of one protein affecting the recruitment of the other (cross-correlation analysis in Supplementary Figure 6C,D). To test if this is due to the biophysical properties of the two fluorophores, we co-expressed each protein with two different tags (mCherry-ALC1 and ALC1-mEGFP, or mCherry-PARP1 and mEGFP-PARP1; Supplementary Figure 7). In the case of ALC1, both fluorophores showed almost identical recruitment profiles (Supplementary Figure 7A,B). Conversely, mEGFP-PARP1 showed slower recruitment with a lower RFI compared to mCherry-PARP1 (Supplementary Figure 7C,D), which may explain a less pronounced difference in recruitment between mEGFP-PARP1 and mCherry-ALC1 (Figure 7f).

Given that endogenous PARP1 may limit access of exogenous PARP1 to DNA damage sites, we further examined relative PARP1 and ALC1 recruitment in PARP1 knock-out (KO) U2OS cells [50]. Indeed, both mCherry-PARP1 and mEGFP-PARP1 recruitment in PARP1 KO cells was faster and more pronounced compared to PARP1 WT cells (compare KO orange and WT red curve in Figure 7b; KO blue and WT green curve in Figure 7f), whereas ALC1 recruitment was slighty increased or did not change (compare KO blue and WT green curve in Figure 7b; KO orange and WT red curve in Figure 7f). These experiments unequivocally demonstrated that PARP1 recruitment precedes ALC1.

Earlier recruitment of PARP1 compared to ALC1 was to be expected considering that ALC1 recruitment to DNA damage sites is known to be PAR-dependent and an ALC1 PAR-binding mutant shows severely impaired recruitment [15,16]. To substantiate the dependence of ALC1 recruitment on PARP1 catalytic activity, we repeated the experiments in PARP1 WT and KO cells using a catalytic mutant of PARP1, E988K, which shows slower accumulation and longer residence at DNA damage sites [17,51] (Supplementary Figure 8). In PARP1 WT cells, ALC1-mEGFP or ALC1-mCherry showed slower recruitment when co-transfected with PARP1 E988K compared to PARP1 WT (Figure 7b, d, f, h). In PARP1 KO cells, ALC1 recruitment was completely impaired in the presence of PARP1 E988K (Figure 7d, h), confirming that PARP1 catalytic activity is a prerequisite for ALC1 recruitment.

Collectively, our results underscore the unique value of simultaneous dual-fluorophore imaging for elucidating the recruitment sequence of fast-recruiting proteins.

## Discussion

Laser microirradiation has become an established methodology for studying the recruitment of proteins to DNA damage sites. Microscopy setups based on imaging of a single fluorescently labelled protein population after laser-induced DNA damage allow investigation of the kinetics of a single protein population recruitment to damage sites. Accurate comparison of recruitment kinetics with other proteins may be limited due to high cell-to-cell variability [52]. To overcome this limitation, it is necessary to image proteins within a single cell. One possibility is to image two channels sequentially, which entails reduced temporal resolution. In order to image dual protein recruitment with high temporal resolution, we coupled a widefield microscope with a commercially available wavelength-splitter, allowing simultaneous imaging of two channels by spatially dividing the emission light. Dual-channel imaging allows direct comparison of the recruitment kinetics of two proteins in a single cell.

Analysis of recruitment kinetics relies on fast imaging and robust quantification of changes in fluorescence intensity at the damage site compared to undamaged regions. Most commonly, recruitment is quantified using relative fluorescence intensity (RFI) given by the intensity at each time point (I_t_) divided by the intensity at time point 0 (I_0_). The RFI thus represents the fold change of the initial intensity upon recruitment. Bleaching correction is also included in standard recruitment analysis by dividing fluorescence intensity with the total nucleus fluorescence at each time point. We improved the standard analysis pipeline at multiple levels. In addition to the bleaching and background correction, we calculated the intensity at each time point by using the median of the twenty highest intensities within the damaged region rather than just the mean or the median value. Most importantly, we introduced an additional recruitment parameter, fraction of maximum recruitment (FMR), whereby RFI is rescaled from 0 to 1, 0 being RFI at t_0_ and 1 being maximum RFI. FMR displays the kinetics of protein recruitment independent of the amplitude, which is particulary useful for analyzing relative recruitment kinetics of two proteins.

Analyzing protein recruitment at a single cell level reveals heterogeneity in recruitment kinetics among different cells. Heterogeneity may be partly due to variable expression levels. For example, overexpression of ALC1 was shown to prolongue its retention at damage sites compared to endogenous ALC1 [44]. Our data show some correlation between initial fluorescence intensity, as an indicator of protein expression levels, and the time of maximum recruitment, whereby lower initial intensity leads to faster recruitment. This may be due to increased levels of protein already present at the damage site, reducing the need for fast recruitment to the site. Selecting cells with lower expression levels is particularly important for transient transfections, but also for stably expressed or endogenously tagged proteins, as endogenous protein expression levels may vary up to 1000-fold in a cell population. When analyzing co-recruitment of two proteins, it is important to bear in mind that relative expression levels may mutually affect recruitment kinetics. Our analysis method comprises cell-by-cell analysis of potential effects of expression levels (measured by initial fluorescence intensity) on recruitment strength (maximum fluorescence intensity) and recruitment kinetics (e.g., time at which the maximum or half-maximum RFI is reached). This in-built comparison is a useful tool to distinguish biological effects from potential artefacts introduced by variable expression levels and enables us to robustly compare different protein pairs and different experimental conditions.

Heterogeneity in recruitment kinetics observed among cells may also stem from BrdU, which is used as a presensitizer to lower the energy required to induce strand breaks. Variable efficiency of BrdU incorporation may result in a variable density of strand breaks, which is known to affect the kinetics of protein recruitment to DNA damage sites [3].

The analysis of relative recruitment kinetics of PCNA and PARG using the 355 nm laser microirradiation system with split-view showed that PARG has faster initial recruitment and faster dissociation compared to PCNA. While PARP1 depletion does not affect PCNA recruitment, as shown previously [34,36,53], it decreases PARG recruitment to DNA damage sites [36,37]. Using simultaneous dual-channel imaging we showed that in the absence of PARP1, PARG and PCNA are recruited with a comparable kinetic mechanism at a single cell level.

The added value of simultaneous dual-channel imaging is the analysis of the recruitment sequence of fast-recruting proteins, such as PARP1 and ALC1. The chromatin remodeler ALC1 is rapidly recruited to DNA damage sites in a PAR-dependent fashion [15,16]. PAR binding to the macro domain of ALC1 releases its autoinhibitory effect on ALC1 ATPase activity [54,55]. Mutations of residues in the macro domain that are required for the autoinhibitory effect of the ALC1 macro domain on ALC1 ATPase activity were shown to accelerate ALC1 recruitment to DNA damage sites [55]. Taken together, ALC1 recruitment is expected to occur after PARP1 recruitment. However, given that both PARP1 and ALC1 are recruited rapidly, standard single-channel imaging or standard sequential dual-channel imaging cannot be used to determine their relative recruitment sequence. Simultaneous dual-channel imaging allowed us to show that PARP1 is indeed recruited earlier than ALC1 and that ALC1 recruitment is entirely dependent on PARP1 catalytic activity.

In conclusion, simultaneous dual-channel imaging of protein recruitment to laser-induced DNA damage sites allows comparative analysis of recruitment kinetics of two proteins at a single cell level. Compared to standard single-channel imaging, simultaneous dual-channel imaging has the potential to reveal differences in relative recruitment dynamics of two proteins, which would otherwise be masked due to averaging of cell populations, and is particularly useful for studying fast-recruiting proteins.

## Materials and methods

### Cell culture

U2OS WT and PARP1 KO [50] cells were maintained in Dulbecco’s Modified Eagle’s Medium (DMEM 4.5 g/l glucose) (Sigma) supplemented with 10% fetal bovine serum (Sigma), 1% L-glutamine (Sigma), 1% penicillin-streptomycin (Sigma) under 5% CO_2_ at 37°C. Transfections were performed with polyethylenimine (PEI; Polysciences). siRNA transfections were performed using Lipofectamine RNAiMax (Ambion, Life Technologies) according to manufacturer’s instructions. siRNA against PARP1 (5ʹ-GCAGCTTCATAACCGAAGAtt-3ʹ) was purchased from Ambion (Silencer® Select). ON-TARGETplus SMARTpool siRNA against PCNA (L-003289–00–0005) was obtained from Dharmacon. Both siRNAs were used at a final concentration of 50 nM. Cells were assayed 72 h after transfection. The level of RNAi knock-down was determined by Western blotting (Supplementary Figure 1).

### Plasmids

mRFP-PCNA was from Cristina Cardoso [56] and mCherry-ALC1 from Sébastien Huet [23]. PARG and PARP1 cDNA were transferred from pDONR221 to pDEST C-mEGFP and pDEST C-tagRFP respectively (from Daniel Gerlich). PARP1 was cloned into mEGFP and mCherry IRES puromycin vectors (Clontech) between AgeI and NotI for N-terminal tagging. PARP1 E988K catalytically dead mutant was generated by site-directed mutagenesis. The following amounts of plasmids were used for double plasmid transfection: 900 ng PARG-mEGFP with 100 ng mRFP-PCNA, 900 ng PARG-mEGFP with 200 ng PARP1-tagRFP, 300 ng mEGFP-PARP1 or 320 ng mEGFP-PARP1 E988K with 100 ng mCherry-ALC1, 80 ng ALC1-mEGFP with 320 ng mCherry-PARP1 or 350 ng mCherry-PARP1 E988K, 320 ng mEGFP PARP1 with 380 ng mCherry-PARP1, 80 ng ALC1-mEGFP with 100 ng mCherry-ALC1.

### Antibodies

The following antibodies were used for immunofluorescence: rabbit anti-PAR polyclonal (Trevigen 4336-BPC-100) and mouse anti-γH2AX (Millipore JBW301). The following antibodies were used for Western blotting: rabbit anti-PARP1 (1:2000; Cell Signaling 9542), rabbit anti-PCNA (1:1000; abcam ab18197) and mouse anti-α-tubulin (1:5000; Sigma Aldrich T6074).

### Immunofluorescence

1.5x10^5^ U2OS cells were seeded on fibronectin coated (1 µg/ml) 35 mm glass-bottom dishes (175 µm ± 15 µm; Greiner Bio-One) 24–48 h before transfection with 300 ng of mEGFP-PARP1 and pre-sensitization with BrdU (10 µM). Cells were damaged at indicated times before fixing and their positions recorded. Cells were fixed with 4% formaldehyde in PBS for 10 minutes while the dish remained inside the microscope on a stable stage to allow locating the damaged cells in their recorded positions. Temperature was maintained at 37°C throughout the IF staining to prevent drifting of the focus. Fixed cells were washed twice in PBS for 5 minutes, permeabilized with 0.5% Triton X-100 in PBS for 8 minutes and washed three times with PBS. Cells were blocked in 0.1% Triton X-100 and 1% BSA in PBS for 20 min. Primary antibodies were diluted 1:500 in blocking buffer and incubated for 30 min after which the cells were washed three times with PBS. Secondary goat anti-rabbit 647 Alexa Fluor® and goat anti-mouse 568 Alexa Fluor® antibodies (Life Technologies) were used at 1:500 dilutions in PBS for 30 minutes. Prior to image acquisition the cells were washed twice with PBS and maintained in PBS. Image acquisition was performed on Zeiss Axio Observer inverted microscope equipped with a Yokogawa CSU-X1-A1 Nipkow spinning disc unit (Visitron Systems; pinhole diameter 50 μm, spacing 253 μm), sCMOS camera (Pco.edge 4.2) camera and an EC Plan-NeoFluor 100x/1.30 NA oil objective (Zeiss). The images were acquired with three laser diodes emitting at 488 nm (set to 88%), 561 nm (set to 100%) and 640 nm (set to 100%) with exposure times of 100 ms.

### Laser microirradiation split-view live imaging widefield microscopy

1.5x10^5^ U2OS cells were seeded on fibronectin coated (1 µg/ml) 35 mm glass-bottom dishes (175 µm +/- 15 µm; Greiner Bio-One) 24 h before transfection. The cells were transfected with plasmids 24–48 h prior to imaging and presensitized with 10 µM 5-bromo-2ʹ-deoxyuridine (BrdU; Sigma) 16 h prior to imaging. Acquisition was performed using a Zeiss Axio Observer inverse widefield microscope equipped for laser-induced microirradiation with a Roper iLASpulse module (355 nm passively Q-switched pulsed laser, 16 mW average power, 0.8 μJ/pulse, repetition rate 21kHz, pulse width 400 ps). An environmental chamber system (PECON) allows stable conditions for temperature and CO2. An EC Plan-NeoFluor 100x/1.30NA objective was used for imaging. Excitation was carried out using a Spectra-X light engine. For simultaneous imaging excitation LEDs for GFP and RFP, filtered by a dual-band 468/553 filter and a single-band 554/23 filter (Semrock) were used respectively. The beam splitter was a dichroic ZT405/488/561/640rpc (Chroma). For PARG-PARP1 and PARG-PCNA UV laser intensity was set to 20% and laser pulse duration was 5 ms/pixel along a stripe length of 11 µm. For PARP1-ALC1 UV laser intensity was set to 80% and laser pulse duration was 0.3 ms/pixel along a stripe length of 6.6 µm. 20% and 80% laser intensity generate 130 and 820 μW power at the sample respectively. For simultaneous emission the Optosplit II setup (Cairn research) was used. The splitting cube hosted a Chroma 512/42m, T570lpxr and Chroma ET570lp set of filters. For emission clean-up LP515 and BP/605/70 filters were used for detecting green and orange dye emissions respectively. An sCMOS camera (Pco.edge 4.2) was used for detection. Microscope control and image acquisition was performed using (Visitron Systems, version 3.1.0.5). Standard acquisition sequence contained 600 time points at an interval of 0.5 s. Channels were simultaneously acquired at 100 ms exposure time for a single slice. For Figure 7: 600 time points with 0.1 s interval and 50 ms exposure time. An additional z-stack was acquired after the time-lapse in order to ensure that the position of maximum recruitment remained in focus during the complete acquisition. Cells were individually chosen according to a stringent fluorescence intensity range to minimise residual GFP bleed-through effects (RFP intensity>GFP intensity, GFP intensity range: 140–200, RFP intensity range: 200–400). Only cells in G1/2 phase were analysed after preselection according to the absence of S-phase-specific replication foci typical for PARG and PCNA. Fiji was used for image analysis [57] and MATLAB for data processing using a custom written script (provided as Supplementary material). Time-lapse figures were created with OMERO.figure [58].
