# Supplementary material for: Simultaneous dual-channel imaging to quantify interdependent protein recruitment to laser-induced DNA damage sites
Source: Nucleus. 2018 Oct 20;9(1):474–91. doi: 10.1080/19491034.2018.1516485 (PMC6284507; doi:10.1080/19491034.2018.1516485)
Supplement: Supplemental Material [file kncl-09-01-1516485-s001.pdf]

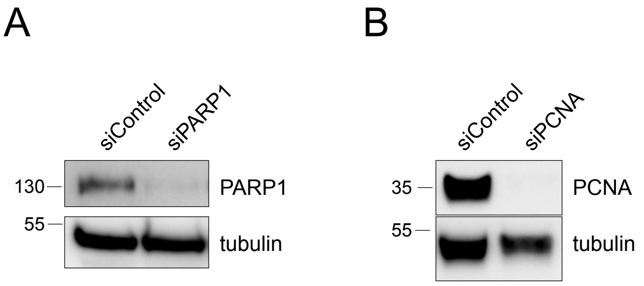

Supplementary Figure 1. Western blot analysis of siRNA-mediated PARP1 and PCNA depletion in U2OS cells. Lysates from cells used for imaging and transfected with siControl and A) siPARP1 or B) siPCNA were analyzed by Western blotting using anti-PARP1 and anti-PCNA antibodies, and anti-α-tubulin as a loading control.

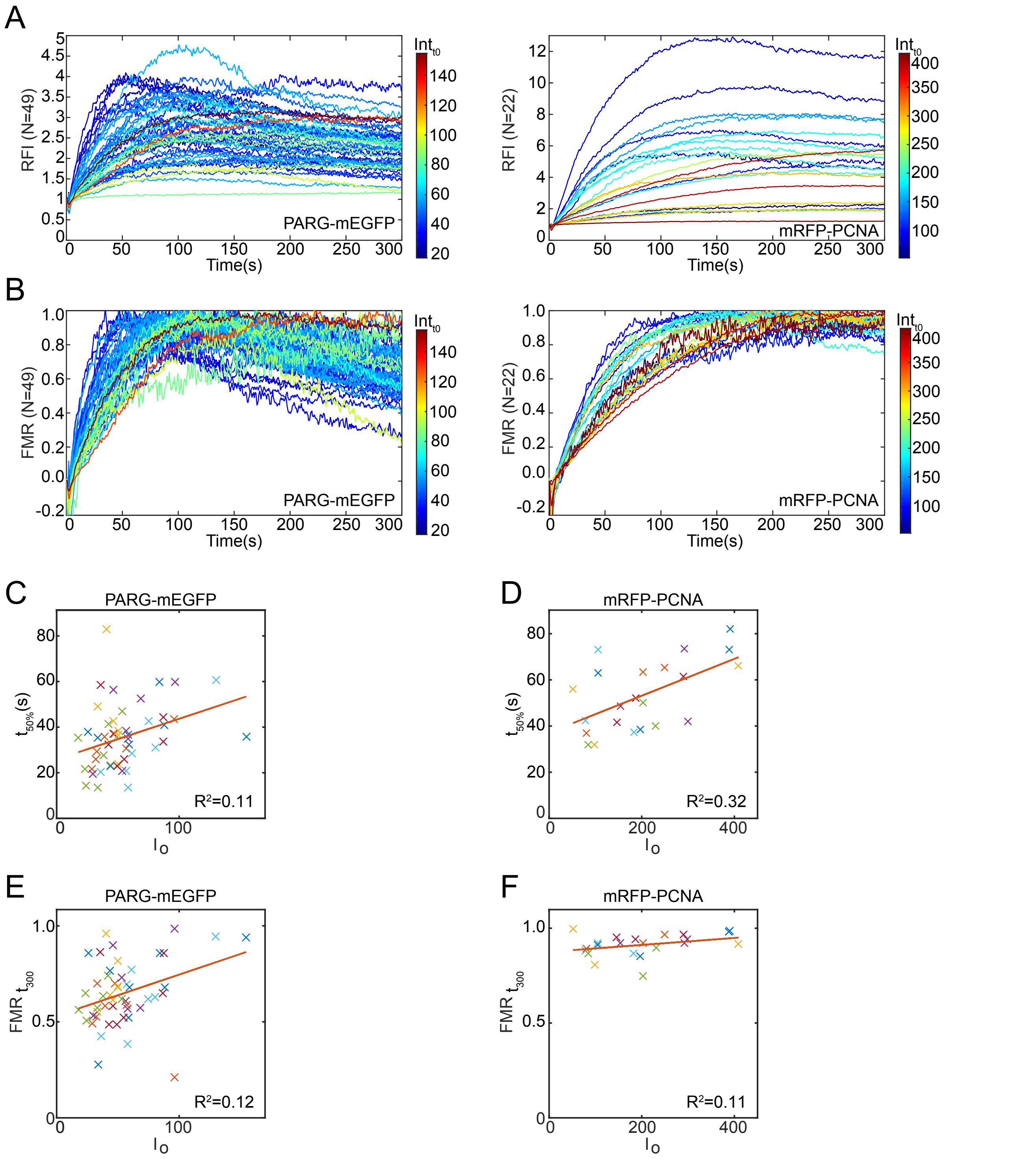

Supplementary Figure 2. The effect of initial fluorescence intensity on PARG and PCNA recruitment after single plasmid transfection. A) Relative fluorescence intensity (RFI) of PARG-mEGFP (left panel) and mRFP-PCNA (right panel). Individual cells are colour coded according to initial intensity. B) Fraction of maximum recruitment (FMR) of PARG-mEGFP (left panel) and mRFP-PCNA (right panel). C,D) Scatter plots showing the time point when 50% of maximum RFI was reached relative to the initial fluorescence intensity for C) PARG-mEGFP and D) mRFP-PCNA. E,F) Scatter plots showing the fraction of maximum recruitment (FMR) after 300 seconds relative to the initial fluorescence intensity for E) PARG-mEGFP and F) mRFP-PCNA. R^2 values determined by linear regression analysis are indicated on the plots.

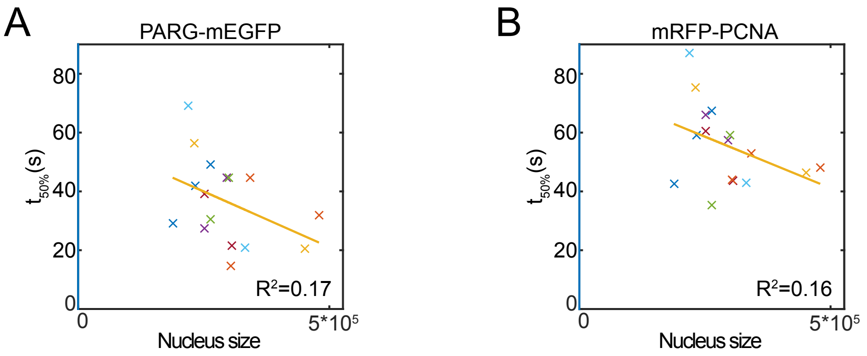

Supplementary Figure 3. Nuclear size does not affect PARG and PCNA recruitment profiles. Scatter plots showing the time point when 50% of maximum RFI was reached relative to the size of each nucleus for A) PARG-mEGFP and B) mRFP-PCNA. The outline of the nuclei was manually defined using FIJI and the nuclear area was measured. R^2 values determined by linear regression analysis are indicated on the plots.

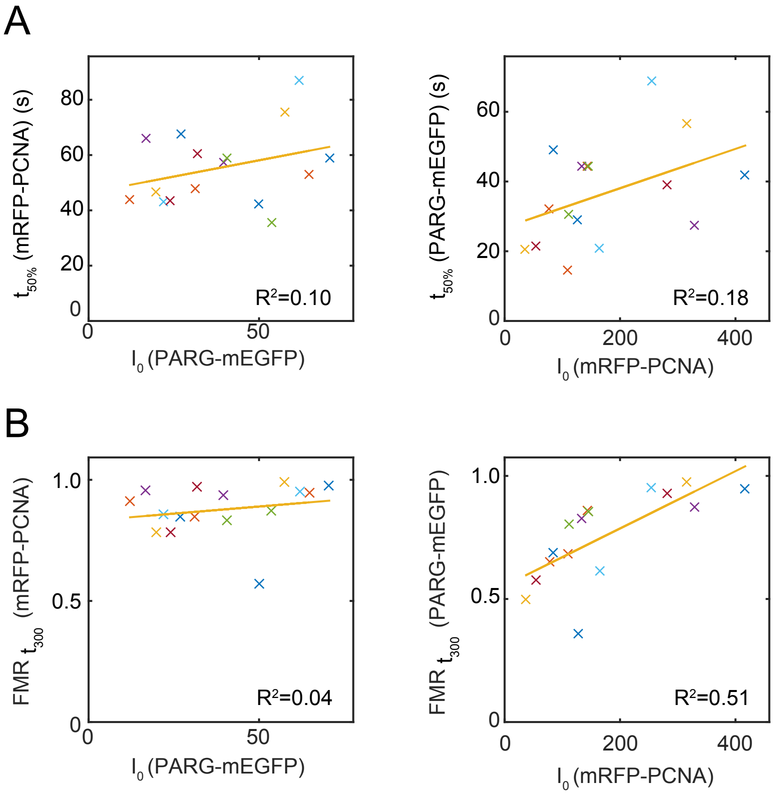

Supplementary Figure 4. Cross-correlation analysis of the recruitment of one protein relative to the initial intensity of the other for PARG and PCNA. A) Scatter plots showing the time point when 50% of maximum RFI was reached for one protein relative to the initial fluorescence intensity of the other. B) Scatter plots showing the fraction of maximum recruitment (FMR) after 300 seconds relative to the initial intensity. R^2 values determined by linear regression analysis are indicated on the plots.

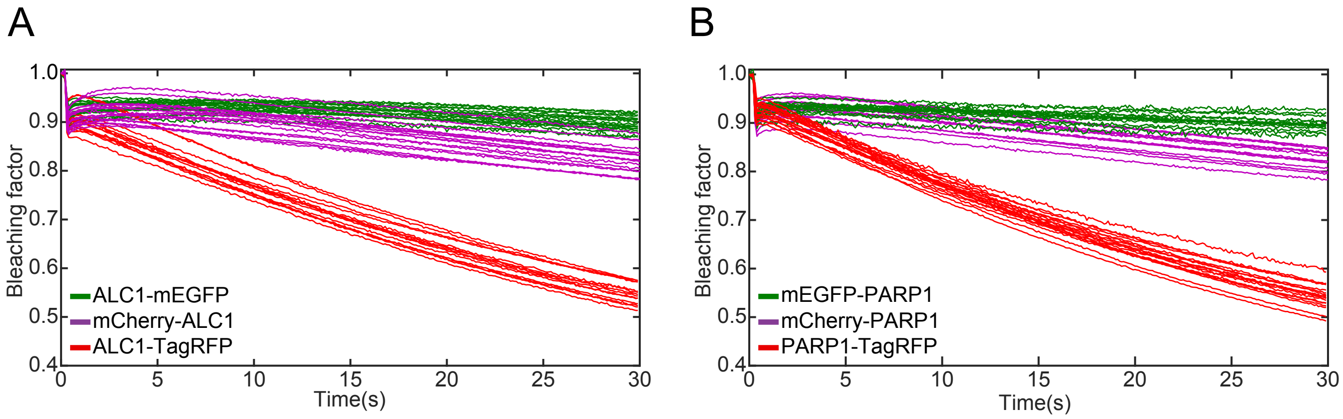

Supplementary Figure 5. **mEGFP and mCherry fluorophores exhibit comparable bleaching behaviour.** The bleaching factor for A) ALC1-mEGFP, mCherry-ALC1 and ALC1-TagRFP, and B) mEGFP-PARP1, mCherry-PARP1 and PARP1-TagRFP is shown. The bleaching factor is the ratio of the mean intensity of the whole nucleus ROI at time point zero and the mean intensity at time point x. The top-twenty median (medMax) is subsequently divided by the bleaching factor to correct for the individual properties of different fluorescently-tagged proteins.

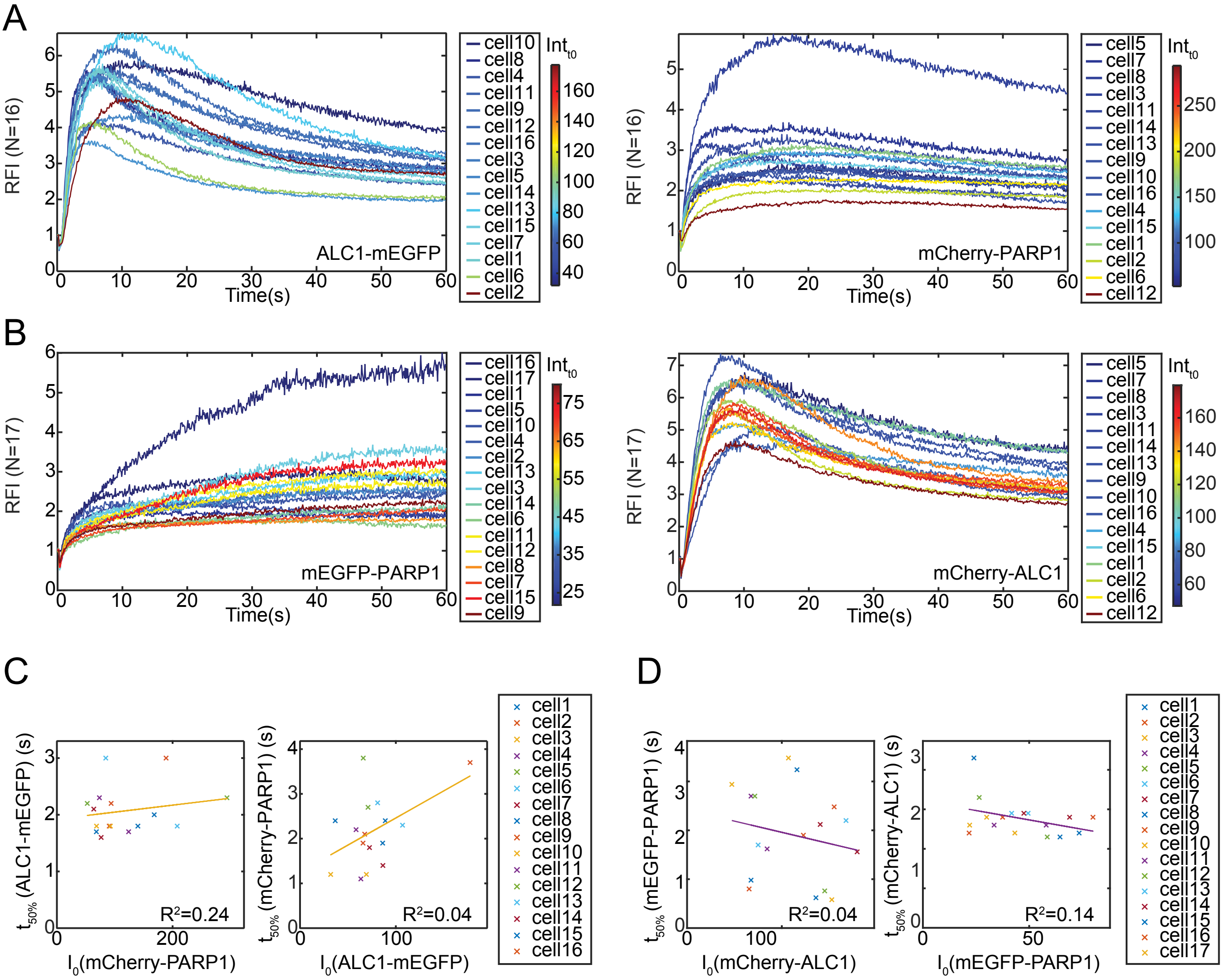

Supplementary Figure 6. Recruitment profiles of differently tagged PARP1 and ALC1 for individual cells and cross-correlation analysis of recruitment of one protein relative to the initial intensity of the other. Relative fluorescence intensity (RFI) of A) ALC1-mEGFP (left panel) and mRFP-PCNA (right panel), and B) mEGFP-PARP1 (left panel) and mCherry-ALC1 (right panel). Individual cells are colour coded according to initial intensity. C,D) Scatter plots showing the time point when 50% of maximum RFI was reached for one protein relative to the initial fluorescence intensity of the other. R^2 values obtained by linear regression analysis are indicated on the plots.

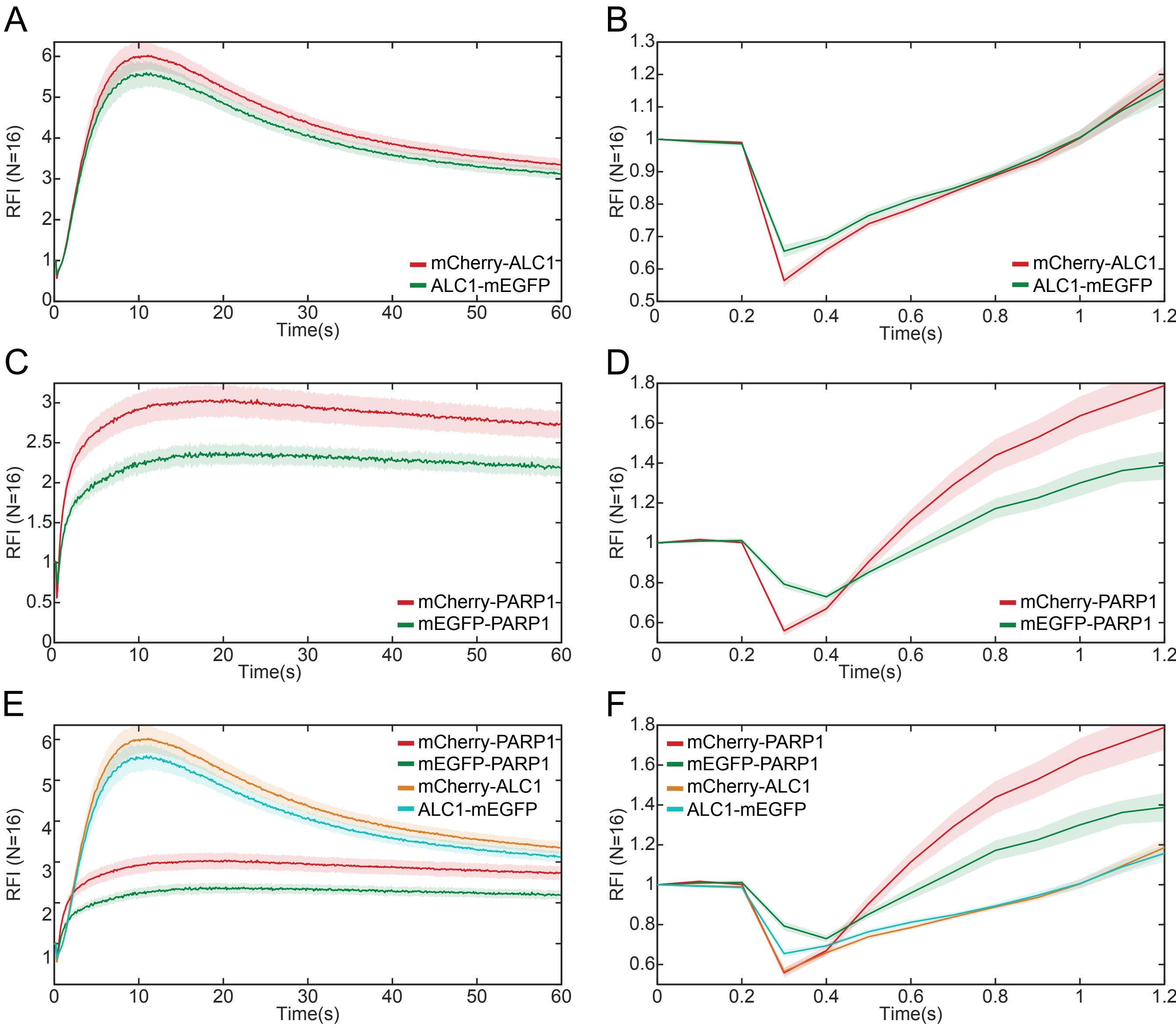

Supplementary Figure 7. The effect of different fluorophores on ALC1 and PARP1 recruitment. Relative fluorescence intensity (RFI) of A,B) mCherry-ALC1 and ALC1-mEGFP, C,D) mCherry-PARP1 and mEGFP-PARP1, E,F) all four proteins combined during A,C,E) 60 s or B,D,F) 1.2 s of imaging after laser-induced DNA damage. Image processing was performed without smoothing. Error bars represent mean +/- SEM.

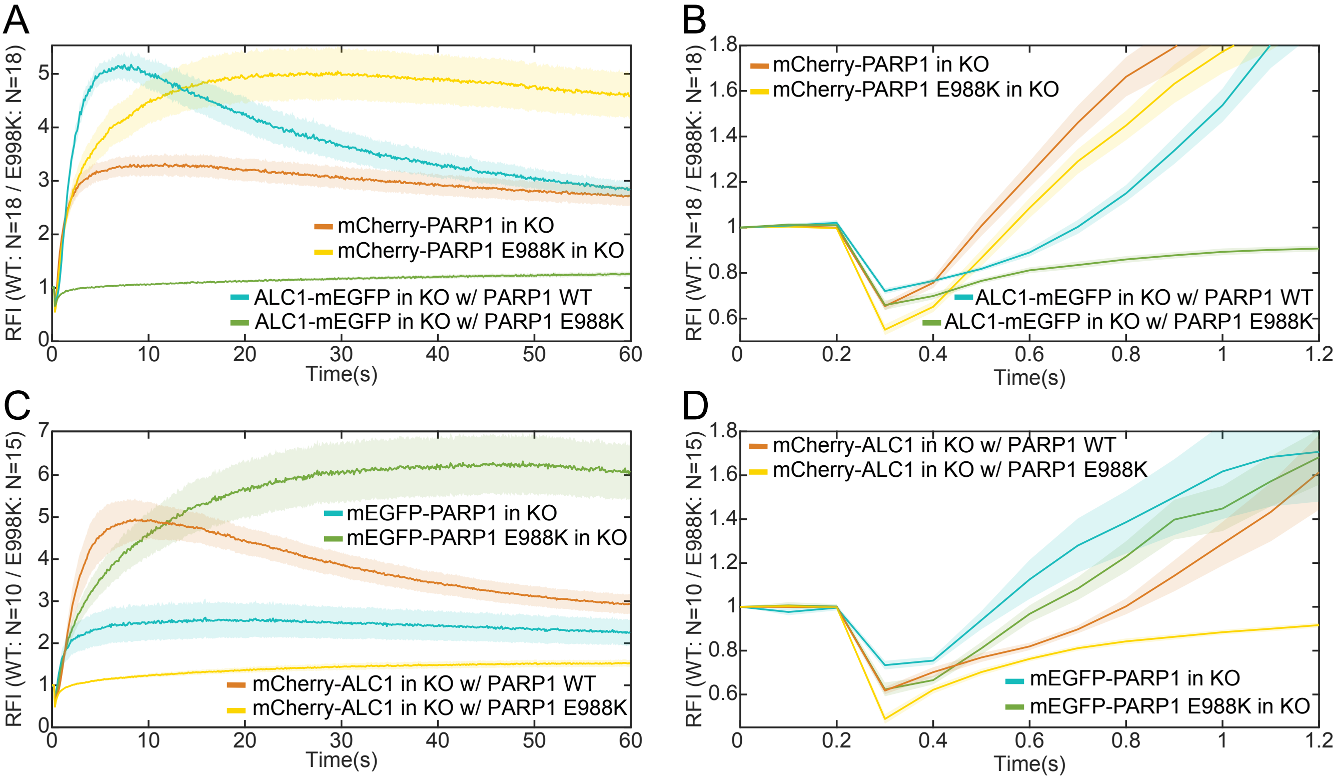

Supplementary Figure 8. PARP1 E988K catalytic mutant is stabilized at DNA damage sites. Relative fluorescence intensity (RFI) of A,B) mCherry-PARP1 WT/E988K and ALC1-mEGFP and C,D) mEGFP-PARP1 WT/E988K and mCherry-ALC1 during A,C) 60 s or B,D) 1.2 s of imaging after laser-induced DNA damage in PARP1 KO U2OS cells. Image processing was performed without smoothing. Error bars represent mean +/- SEM.
